# Blocking Nerve Growth Factor Signaling Reduces the Neural Invasion Potential of Pancreatic Cancer Cells

**DOI:** 10.1371/journal.pone.0165586

**Published:** 2016-10-28

**Authors:** Aditi A. Bapat, Ruben M. Munoz, Daniel D. Von Hoff, Haiyong Han

**Affiliations:** Clinical Translational Research Division, Translational Genomics Research Institute, Phoenix, Arizona, United States of America; The University of Texas MD Anderson Cancer Center, UNITED STATES

## Abstract

Perineural invasion (PNI) is thought to be one of the factors responsible for the high rate of tumor recurrence after surgery and the pain generation associated with pancreatic cancer. Signaling via the nerve growth factor (NGF) pathway between pancreatic cancer cells and the surrounding nerves has been implicated in PNI, and increased levels of these proteins have been correlated to poor prognosis. In this study, we examine the molecular mechanism of the NGF signaling pathway in PNI in pancreatic cancer. We show that knocking down NGF or its receptors, TRKA and p75NTR, or treatment with GW441756, a TRKA kinase inhibitor, reduces the proliferation and migration of pancreatic cancer cells *in vitro*. Furthermore, pancreatic cancer cells migrate towards dorsal root ganglia (DRG) in a co-culture assay, indicating a paracrine NGF signaling between the DRGs and pancreatic cancer cells. Knocking down the expression of NGF pathway proteins or inhibiting the activity of TRKA by GW441756 reduced the migratory ability of Mia PaCa2 towards the DRGs. Finally, blocking NGF signaling by NGF neutralizing antibodies or GW441756 inhibited the neurite formation in PC-12 cells in response to conditioned media from pancreatic cancer cells, indicating a reciprocal signaling pathway between the pancreatic cancer cells and nerves. Our results indicate that NGF signaling pathway provides a potential target for developing molecularly targeted therapies to decrease PNI and reduce pain generation. Since there are several TRKA antagonists currently in early clinical trials they could now be tested in the clinical situation of pancreatic cancer induced pain.

## Introduction

Pancreatic cancer is one of the most deadly types of cancer and is the fourth leading cause of cancer related deaths in the US. Despite recent advances in the diagnosis and treatment of pancreatic cancer, prognosis for pancreatic cancer patients is rather bleak given that the 5-year survival rate is less than 6% [1,2]. One of the reasons for this dismal prognosis is due to the high rate at which the tumor recurs despite surgical resection of the tumor [3,4,5]. A potential reason for the high rate of relapse has been postulated to be the ability of the pancreatic cancer cells to invade the surrounding nerves in a process known as perineural invasion (PNI). The close proximity of the pancreas to a number of neural plexuses and the rich innervations into the pancreas may be a reason for this increased incidence of PNI [6,7]. Initial theories suggested that the pancreatic cancer cells traveled along a plane of least resistance [3,8], but recent advances have identified it to be a highly coordinated process involving a number of signaling molecules secreted by both the nerves and the pancreatic cancer cells, generating a mutual tropism between them [9,10,11,12]. Further, upon invading the nerves, the pancreatic cancer cells were thought to be part of a privileged environment which allowed them to survive and prosper within the neuronal spaces [11,13,14]. The invasion of nerves by the pancreatic cancer cells also damages the nerve ends and exposes them to noxious stimuli, resulting in pain experienced by pancreatic cancer patients [4,5,13,15].

Nerve growth factor (NGF) has two known receptors: the specific, high-affinity receptor, tropomyosin-receptor kinase A (TRKA), which has been reported to activate pro-survival signaling in the pancreatic cancer cells and nerves, and the low-affinity receptor, p75 neurotrophin receptor (p75^NTR^), which also binds other neurotrophins [16]. Elevated levels of NGF in pancreatic cancer cells and high levels of its receptors, TRKA and p75^NTR^ in the surrounding nerves, have been correlated to poor prognosis and PNI in pancreatic cancer patients [17,18,19,20]. Absence of TRKA in nerve cells results in NGF binding to p75^NTR^ and leads to activation of pro-apoptotic signals [16,21]. In pancreatic cancer cells in the presence of TRKA, p75^NTR^ enhances binding of NGF to TRKA and results in increased signaling via the NGF-TRKA pathway [16,21]. Thus, elucidating the molecular mechanism of NGF signaling via its receptors, TRKA and p75^NTR^ in pancreatic cancer cells would help decipher the mechanism of PNI in pancreatic cancer.

## Materials and Methods

### Cell culture

Pancreatic cancer cell lines BxPC-3 and Mia PaCa2 obtained from the American Type Culture Collection (ATCC) (Manassas, VA) were cultured in RPMI-1640 medium (Invitrogen, Carlsbad, CA) supplemented with 10% Fetal Bovine Serum (FBS) (Gemini Bio-Products, Woodland, CA) and 1% Penicillin-Streptomycin (Invitrogen, Carlsbad, CA). Rat pheochromocytoma cells, PC-12 cells (obtained from ATCC) were cultured in suspension in RPMI-1640 media (Invitrogen, Carlsbad, CA) with 5% FBS, 10% Horse Serum (Gemini Bio-Products, Woodland, CA) and 1% Penicillin-Streptomycin. Cell line identities were verified by short tandem repeat (STR) profiling (16) using the AmpFISTR Identifiler PCR amplification Kit (Applied Biosystems/Thermo Fisher Scientific, Waltham, MA). Results were compared with published STR sequences from the ATCC. The STR profiling is repeated once a cell line has been passaged more than 6 months after previous STR profiling.

### Sulforodamine B (SRB) cell proliferation assay

The effect of a TRKA inhibitor, GW441756 (Tocris Biosciences, Minneapolis, MN) on the growth and survival of Mia PaCa2, and BxPC-3 pancreatic cancer cell lines was determined using the sulforodamine B (SRB) assay [22,23]. 3,000 cells of each cell line were plated in each well of a 96-well plate in triplicate in a 90μl volume and allowed to grow overnight. A 10 point, 2-fold serial dilution of GW441756 starting from 100μM (final concentration) was used and each dose was added to the wells in a 10 μl volume to get a final volume of 100 μL. After 72 hours of incubation, the media from the cells was aspirated off and 65μl of 10% trichloroacetic acid (TCA) was added to the wells and incubated at 4°C for 30 minutes to fix the cells. The plates were then washed 5 times with dH_2_O and allowed to dry at room temperature for 15 minutes. 40μl of 0.04% SRB dye dissolved in 1% acetic acid was added to the wells and allowed to incubate at room temperature for 30 minutes. The plates were washed 5 times with 1% acetic acid and allowed to dry at room temperature for 15 minutes. To dissolve the precipitate SRB formed, 100μl of 50mM Tris buffer was added and the plates were placed on a shaker for 20 minutes at room temperature. The absorbance was measured at 570nm using a plate reader (BioTek, Winooski, VT) [22,23,24]. The assay was performed in triplicate and repeated at least three times.

### siRNA transfection of Mia PaCa2 and BxPC-3 cells

Approximately 1.5×10^5^ Mia PaCa2 or BxPC-3 cells were plated in each well of a 6-well plate in serum free media (SFM) and allowed to attach overnight. On the next day, siLentFect reagent (BioRad, Hercules, CA) was used to deliver the SMARTPool siRNA (Dharmacon RNAi Technologies, Thermo Scientific, Pittsburg, PA) for NGF, p75^NTR^ and TRKA at a 100nM concentration. A non-targeting siRNA as a negative control and a siRNA against the UBB protein as a positive control for transfection were also transfected into the cells using the manufacturer’s recommended protocol. Briefly, 4μl of siLentFect and 100nM siRNA were mixed together in equal volumes, in serum free medium (SFM) in poly-styrene tubes and allowed to incubate at room temperature for 30 minutes. All the media from the wells was aspirated off and 500 μl of the siRNA—siLentFect mix was added to each well along with 1.5ml of media. After incubation for 72 hours cells were assayed for NGF, p75^NTR^ and TRKA protein expression and used in the Boyden Chamber migration assay and Dorsal root ganglion co-culture assay.

### Gene expression analysis using Western blotting and RT-PCR

Levels of NGF, p75^NTR^ and TRKA proteins in Mia PaCa2 and BxPC-3 cells treated with siRNA were determined using Western blotting. siRNA treated cells were lysed in RIPA buffer (50 mM Tris-HCl, pH 8.0, 150 mM NaCl, 1.0% Igepal CA-630 (NP-40), 0.5% sodium deoxycholate, and 0.1% SDS) and the amount of protein in the cell extracts was quantified using the BCA assay (Thermo Scientific, Pittsburg, PA). Forty-five micrograms of protein was loaded onto a 4–12% Bis-Tris pre-cast gel (Invitrogen, Carlsbad, CA) and allowed to separate at 200V for 45 minutes. The gel was then transferred onto a nitro-cellulose membrane at 30V for 1 hour at room temperature. Following the transfer of proteins, the membranes were blocked in 5% blocking solution made from, non-fat dry milk dissolved in 1x TBST (1×TBS + 0.1% Tween 20) for 1–3 hours. Antibodies to NGF (Epitomics, Burlingame, CA), p75^NTR^ (Santa Cruz Biotechnology, Dallas, TX) and TRKA (Santa Cruz Biotechnology) all at a dilution of 1:200 were added to the respective membranes in 5% blocking solution overnight at 4°C. The next day the membranes were washed with 1× TBST twice for 10 minutes and the anti-rabbit HRP conjugated secondary antibody (Cell Signaling Technology, Danvers, MA) was added to the blots at a dilution of 1:1000 for 1–2 hours at room temperature and then washed in 1× TBST four times for 10 minutes. The membranes were developed using the ECL chemiluminescent substrate (Millipore, Billerica, MA) and were visualized and quantitated with the Bio Spectrum 500 Imaging System (UVP, Cambridge, UK). The blots were also processed as described above for the detection of β-Actin which was used as an internal loading control. The actin antibody (Sigma-Aldrich, St. Louis, MO) was used at a dilution of 1:1000 along with the anti-mouse secondary antibody (Cell Signaling Technology) also at a dilution of 1:1000. All experiments were repeated three times.

Cells treated with siRNA oligonucleotides were also harvested for RT-PCR analysis of mRNA expression. Total RNA extraction was extracted using the RNeasy Mini Kit (Qiagen) was utilized following the manufacturer’s recommended protocol. One microgram of total RNA was used in a 20 μl cDNA synthesis reaction (Quantas Biosciences, Gaithersburg, MD). Reactions were carried out in 1 μl of the cDNA reaction mix, 10 μl SYBR Green/Taq Polymerase master mix (Roche, Indianapolis, IN), 4 μl of primers (final concentration 400 nM), and 5 μl water to a final volume of 20 μl. The Bio-Rad MyIQ single color real-time PCR detection system (Hercμes, CA) was used to perform the RT-PCR. Two-step amplification (95°C for 30 sec and 58°C for 30 sec) was repeated for 40 cycles. Following the PCR reaction, a melting curve analysis was performed (60°C for 5 sec) for 35 cycles. The data was analyzed using the comparative CT method [25].

### Boyden chamber migration assay

The migratory ability of Mia PaCa2 and BxPC-3 treated with the TRKA inhibitor, or the siRNA oligonucleotides against NGF, p75^NTR^ and TRKA was determined using the Boyden Chamber migration assay [26]. Five hundred microliters of media with 10% FBS and 10μg/ml fibronectin was placed in the bottom well as a chemoattractant and 5×10^5^ cells/ml solution of treated cells prepared in SFM was placed in the transwell inserts (BD Biosciences, San Jose, CA) and were allowed to migrate at normal tissue culture conditions for 24 hours. The membranes were fixed in 100% methanol and 75% ethanol followed by a wash in water and then stained with Histogene (Life Technologies, Grand Island, NY), a dye that stains both the cytoplasm and nuclei. The membranes were then washed and fixed in 75% ethanol, 95% ethanol and finally in 100% ethanol. The membranes were then dried at room temperature, cut using a scalpel, dipped in xylene and mounted on slides with ~200μl per mount and set overnight in dark drawer. Pictures of the membranes were taken and cells in at least 5 fields were counted. The experiment was done three times.

### PC-12 neurite outgrowth assay

Conditioned medium was collected from Mia PaCa2 and BxPC-3 cells plated in T75 flasks after 10 days and used in the PC-12 neurite outgrowth assay [27]. 15,000 PC-12 cells were plated in a 12-well plate coated with 0.05mg/ml collagen and allowed to adhere overnight. The next day conditioned media from Mia PaCa2 or BxPC-3 cells was added to the cells along with a neutralizing NGF antibody (PeproTech, Rocky Hill, NJ) and the TRKA inhibitor, GW441756, Purified NGF protein (Sigma-Aldrich) was used as a positive control for induction of neurites. Pictures of the cells were taken and at least 5 fields were counted. The experiment was repeated three times.

### Dorsal root ganglion (DRG) co-culture assay

Dorsal root ganglia (DRG) were isolated from Wistar rats (obtained from Taconic Biosciences, Hudson, NY) using a protocol adapted from Malin SA *et al* [28]. The use of rats and the procedure for isolating DRG from the rats for this study were reviewed and approved by the Institutional Animal Care and Use Committee (IACUC) of the Translational Drug Development (TD2) where the animal work was performed (Protocol #12008). Briefly, Wistar rats were euthanized and prepared for isolation of dorsal root ganglia by clipping the hair on the rat’s dorsal surface and sterilized with 70% ethanol. From the dorsal side, the vertebral column and then the spinal cord were exposed. Dorsal root ganglia were isolated from the lumbar, thorasic and cervical regions and placed in tissue culture media containing antibiotics for use in the DRG co-culture assay (adapted from [9,10]). GFP labeled Mia PaCa2 cells were pre-treated with siRNA to NGF, p75^NTR^, TRKA for 72 hours and with neutralizing NGF antibody (PeproTech, Rocky Hill, NJ) and the TRKA inhibitor, GW441756 for 48 hours. The pre-treated cells were trypsinized and counted and cell suspension (5 × 10^6^ cells/ml) was prepared in Matrigel (BD Biosciences) on ice. Individual DRG’s were placed in a 12-well tissue culture plate and a 20μl drop of Matrigel was placed on it and allowed to congeal at 37°C for 15 minutes. A 25μl drop of the cell suspension in Matrigel is placed adjacent to it touching the drop of Matrigel containing the DRG. The plates were placed at 37°C for 15 minutes to allow the Matrigel to solidify and then flooded with 2ml of warm media containing 10% FBS and 1% P/S. The ability of the GFP-Mia PaCa2 cells to travel towards the neurites extended from the DRG’s was determined on Day 10. The assay was done five or more times and the data is presented as average with standard error of the individual experiments. The invaded GFP-Mia PaCa2 cells were calculated as a relative neural invasion index [29], by measuring the distance travelled by the GFP-Mia PaCa2 cells divided by the total distance between the DRG and the GFP-Mia PaCa2 cells. The distances were measured using the ImageJ software to calculate the relative invasion index.

### Statistical Analysis

To compare the effects of either siRNA or drug treatment on the pancreatic cancer cells in each of the assays, Student’s *t*-test was used to calculate the p value between different groups as indicated in the figure legends.

## Results and Discussion

### Effect of knocking down the expression of NGF-TRKA signaling pathway proteins on the survival and migration of pancreatic cancer cells

**Fig 1:**
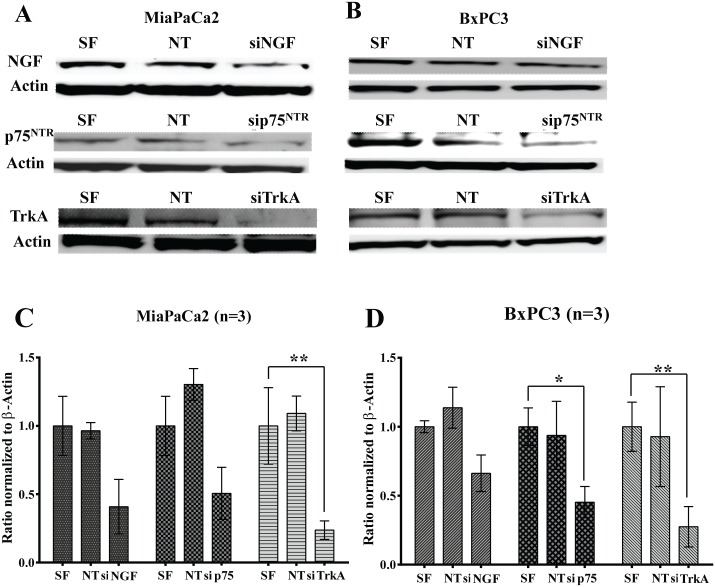
Knocking down the expression of the NGF-TRKA signaling pathway genes using siRNA oligonucleotides. The cells were treated with the siRNA oligonucleotides for 72 hours and then subjected to Western blotting (A and B) or RT-PCR analysis (C and D) for NGF, p75^NTR^ and TRKA expression in Mia PaCa-2 (A and C) or BxPC-3 (B and D) cells. SF: siLentFect (transfection reagent), NT: Non-targeting siRNA control. *: p ≤ 0.05; ** ≤ 0.01.

**Fig 2:**
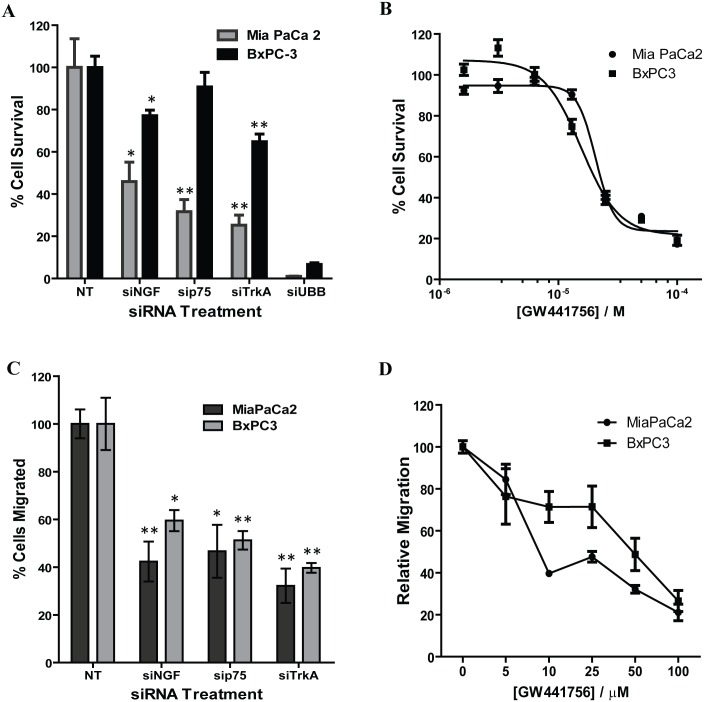
Knocking down the expression of the NGF-TRKA signaling pathway proteins or inhibiting the activity of TRKA reduces the growth and migratory activity of pancreatic cancer cells. A) Cell growth inhibition by siRNA treatment in Mia PaCa-2 and BxPC-3 cells. Cell viability was measure 72 hours post siRNA treatment and compared to Non-targeting (NT) siRNA control. B) Cell growth inhibition by TRKA inhibitor GW441756. Cells were treated with the inhibitor for 72 hours. C) siRNA knockdown of the expression of NGF, p75^NTR^ or TRKA reduced migration of pancreatic cancer cells. D) GW441756 inhibited the migration of pancreatic cancer cells in a dose dependent manner. *: p ≤ 0.05; ** ≤ 0.01 (compared to Non-targeting control).

Signaling via the NGF-TRKA pathway is not only involved in the process of PNI but is also involved in the growth and survival of pancreatic cancer cells [3,8,20,21,29]. To understand the role of the NGF-TRKA signaling pathway in PNI, we knocked down expression of NGF and its receptors TRKA and p75^NTR^ in Mia PaCa2 and BxPC-3 pancreatic cancer cell lines to determine its effect on pancreatic cancer cell survival. Expression of NGF and its receptors TRKA and p75^NTR^ was reduced 72 hours after transfection with SMARTPool siRNA both at the protein (Fig 1A and 1B) and RNA level (Fig 1C and 1D) as measured by Western blotting and quantitative reverse transcriptase polymerase chain reaction (qRT-PCR), respectively. Inhibiting the NGF-TRKA signaling pathway reduced survival of both Mia PaCa2 and BxPC-3 cells as measured by the SRB viability assay (Fig 2A). Furthermore, treatment of Mia PaCa2 and BxPC-3 cells with GW441756, an inhibitor of TRKA [30], also reduced viability of the pancreatic cancer cells (Fig 2B). Small molecule TrkA inhibitors have been shown to inhibit the phosphorylation of ERK1/2 via the Ras-Raf-Mek signaling pathway [31]. We also observed the inhibition of ERK1/2 phosphorylation as shown in the S1 Fig. These results indicate that the NGF signaling pathway may be involved in conferring a growth and survival advantage to the pancreatic cancer cells.

To determine whether NGF signaling pathway affects the migration of pancreatic cancer cells, we determined the ability of pancreatic cancer cells to migrate in a Boyden chamber assay after blocking the NGF-TRKA signaling pathway. Both Mia PaCa2 and BxPC-3 cells showed decreased migration when treated with siRNA oligonucleotides targeting NGF, TRKA or p75^NTR^ (Fig 2C). Inhibition of TRKA with GW441756 also reduced the migration of the Mia PaCa2 and BxPC-3 cells in a concentration dependent manner (Fig 2D). In order to control for the growth inhibitory effects of GW441756 treatment, equal numbers of cells were seeded in each chamber and the decreased migratory ability of the pancreatic cancer cells is due to inhibition of signaling from the NGF-TRKA pathway and not due to reduced number of cells. Thus, the NGF signaling pathway is not only involved in the survival of pancreatic cancer cells, but also plays a role in their ability to migrate.

### Disrupting the NGF-TRKA signaling pathway reduces the ability of pancreatic cancer cells to migrate towards neurites extended from dorsal root ganglia in a Matrigel co-culture assay

**Fig 3:**
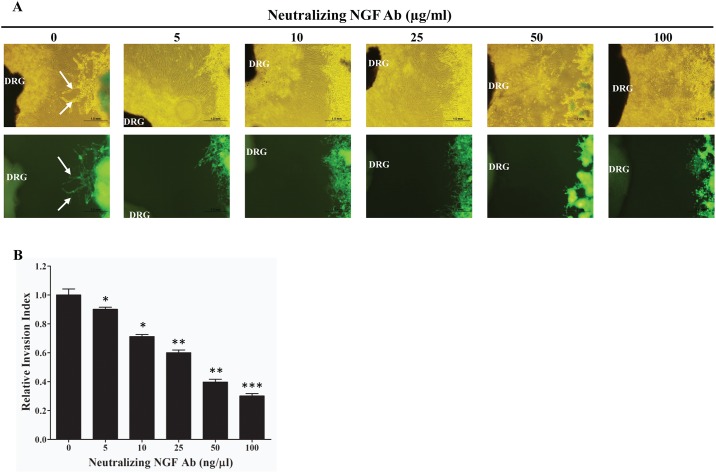
Effect of NGF neutralizing antibody on the ability of pancreatic cancer cells to migrate towards neurites extended from dorsal root ganglia (DRG). A) Representative images (top panel: bright field; bottom panel: green fluorescence) of the migration of Mia PaCa-2 cells towards nerves treated with different concentrations of NGF neutralizing antibody. Arrows indicate the cancer cells that migrated towards the neurites. B) Quantification of the effect on the migration of cancer cells using relative invasion index. *: p ≤ 0.01; ** ≤ 0.001; *** ≤ 0.0001 (compared to no antibody control).

**Fig 4:**
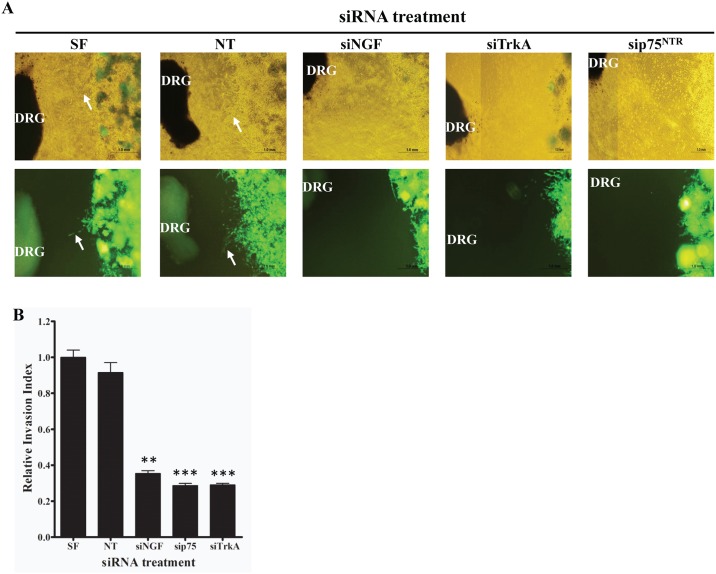
Effect of siRNA knockdown of the expression of NGF, TRKA, or p75^NTR^ on the ability of pancreatic cancer cells to migrate towards neurites extended from DRG. A) Representative images (top panel: bright field; bottom panel: green fluorescence) of the migration of Mia PaCa-2 cells towards nerves treated with siRNA oligonucleotides targeting the indicated genes. Arrows indicate the cancer cells that migrated towards the neurites. B) Quantification of the effect on the migration of cancer cells using relative invasion index. SF: siLentFect (transfection reagent), NT: Non-targeting siRNA control. ** ≤ 0.001; *** ≤ 0.0001 (compared to Non-targeting siRNA control).

**Fig 5:**
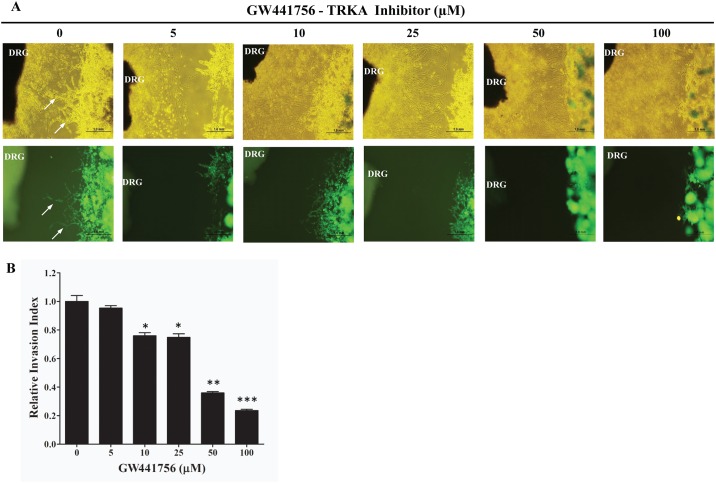
Effect of TRKA inhibitor GW441756 on the ability of pancreatic cancer cells to migrate towards neurites extended from DRG. A) Representative images (top panel: bright field; bottom panel: green fluorescence) of the migration of Mia PaCa-2 cells towards nerves treated with different concentrations of GW441756. Arrows indicate the cancer cells that migrated towards the neurites. B) Quantification of the effect on the migration of cancer cells using relative invasion index. *≤ 0.01; ** ≤ 0.001; *** ≤ 0.0001 (compared to no drug control).

NGF secreted by the surrounding nerves in a paracrine manner may signal via TRKA receptors on the cancer cells to increase their growth and migration towards the surrounding nerves. In order to test whether pancreatic cancer cells migrate towards the surrounding nerves and whether the NGF signaling pathway was responsible, we set up a Matrigel dorsal root ganglion (DRG) co-culture assay [9,10]. GFP labeled Mia PaCa2 (GFP-Mia PaCa2) cells were co-cultured with rat DRG and the ability of the GFP-Mia PaCa2 cells to travel towards the neurites extended by the DRG was determined. Migration of cells towards the DRG was quantified as a ratio of the distance migrated by the GFP-Mia PaCa2 cells and the total distance between the cancer cells and the DRG [29]. Treating the GFP-Mia PaCa2 with a neutralizing NGF antibody to reduce amount of NGF secreted by the GFP-Mia PaCa2 cells reduced the migration of the cancer cells towards the DRG (Fig 3). Furthermore, knocking down the expression of the NGF-TRKA signaling pathway proteins by siRNA also reduced the migration of GFP-Mia PaCa2 cells towards the DRG (Fig 4). Finally, disruption of the NGF-TRKA signaling pathway by inhibiting TRKA with the GW441756 inhibitor also resulted in decreased migration of the cells towards the DRG (Fig 5). Thus, impaired signaling between the Mia PaCa2 cells and the DRG plays a role in reducing migration of the cancer cells towards the DRG. These results indicate that the paracrine signaling between the cancer cells and the nerves might play an important role in the perineural invasion.

### Reciprocal signaling between the pancreatic cancer cells and nerves may stimulate the growth of nerves

**Fig 6:**
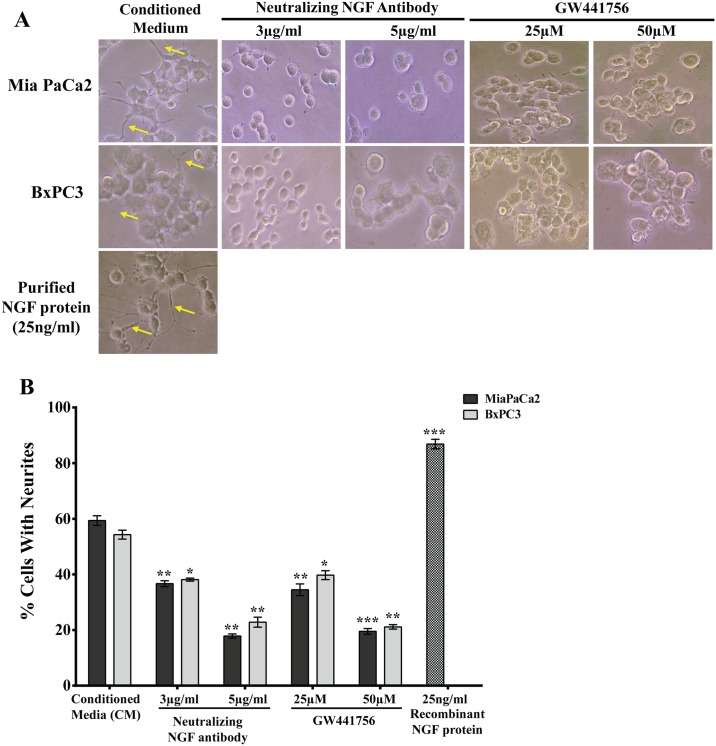
Reciprocal signaling between pancreatic cancer cells and neurites extended from the PC-12 cells is reduced when the NGF-TRKA signaling pathway is disrupted. A) Treatment with the NGF neutralizing antibody or the TRKA inhibitor (GW441756) reduces the neurite outgrowth induced by conditional medium from pancreatic cancer cells. B) Quantification of neurite outgrowth assay shown in A. Conditioned Media (CM) collected from MiaPaCa2 or BxPC3 cells were treated with a neutralizing NGF antibody and a TRKA inhibitor and neurite (yellow arrows) extension from the PC-12 cells was visualized under a bright field microscope. Recombinant NGF protein was used as a positive control. *≤ 0.01; ** ≤ 0.001; *** ≤ 0.0001 (compared to Conditioned Media).

Since inhibition of the NGF-TRKA signaling pathway results in a decrease in the migration of the pancreatic cancer cells towards the neurites, we performed a reciprocal experiment to determine whether inhibition of NGF signaling would reduce the induction of neurites from PC-12 cells. PC-12 cells treated with conditioned media (CM) collected from Mia PaCa2 and BxPC-3 pancreatic cancer cells resulted in neurite induction (Fig 6A). However, addition of a neutralizing NGF antibody or an inhibitor to TRKA, showed a significant reduction of neurites from the PC-12 cells as compared to CM alone and purified NGF protein, which was used as a positive control (Fig 6A and 6B). These results further indicate that targeting the NGF signaling pathway between the pancreatic cancer cells and the surrounding nerves may reduce migration and perineural invasion of pancreatic cancer cells into the nerves.

Perineural Invasion (PNI) is thought to be one of the mechanisms responsible for the high rate of relapse and tumor recurrence [4,5]. PNI is not only an indicator of poor prognosis and decreased survival [4,32], but also generates pain [11,12,16,33] which can further affect the quality of life of pancreatic cancer patients. Signaling via NGF and its receptors TRKA and p75^NTR^ contributes to the process of PNI and elevated levels of NGF and TRKA in the pancreatic cancer cells and increased levels of TRKA and p75^NTR^ in the surrounding nerves have been equated to increased incidence of PNI and pain in pancreatic cancer patients [3,18,20]. In order to understand the molecular mechanisms involved in PNI, we undertook experiments to determine the role that NGF and its receptors play in PNI process. Since the NGF-TRKA signaling pathway is involved in the proliferative and invasive ability of pancreatic cancer cells, reducing levels of NGF and TRKA proteins in Mia PaCa2 and BxPC-3 cells by siRNA reduced their proliferation and migration (Fig 2A and 2C). A similar result was observed when activity of TRKA receptor was inhibited in these same cell lines using a small molecule inhibitor (Fig 2B and 2D). Thus autocrine signaling via the NGF-TRKA signaling pathway in the Mia PaCa2 and BxPC-3 cells increases their invasive and proliferative propensities. In order to determine paracrine signaling via NGF between the pancreatic cancer cells and the surrounding nerves, we set up the dorsal root ganglion (DRG) co-culture assay [9,10]. Untreated GFP-labeled Mia PaCa2 cells grow directionally towards and along the neurites extended by the DRGs (Fig 3). This movement could be the result of autocrine NGF signaling in the GFP-Mia PaCa2 cells and possibly the paracrine signaling between the DRG and GFP-Mia PaCa2 cells. Suppression of NGF using siRNA or a neutralizing antibody reduced the ability of GFP-Mia PaCa2 cells to travel towards the DRGs (Fig 3), which suggests that disrupting the NGF signaling between the DRGs and the cancer cells (GFP-Mia PaCa2) cells could result in a decrease in their invasiveness. Also, down-regulation of p75^NTR^ using siRNA reduced migration of GFP-Mia PaCa2 cells towards the DRGs, implicating a further role for p75^NTR^ in the process of PNI. Expression of p75^NTR^ has been shown to positively correlate to PNI in pancreatic cancer tissues, breast cancer and prostate cancer [34]. Contrary to previous beliefs that expression of p75^NTR^ was inversely correlated to PNI [35], Wang *et al* demonstrated a positive correlation between expression of p75^NTR^ and PNI in pancreatic cancer tissues and also showed that p75^NTR^ was involved in the chemotaxis of pancreatic cancer cells [19]. However, a decrease in the migratory ability of the GFP-Mia PaCa2 cells due to inhibition of NGF and its receptors may solely be a result of autocrine NGF signaling in the GFP-Mia PaCa2 cells. To determine if paracrine NGF signaling is involved in PNI, we examined at the neuritogenesis of PC-12 rat pheochromocytoma cells in response to NGF secreted by Mia PaCa2 and BxPC-3 pancreatic cancer cells (Fig 4). Conditioned media collected from untreated Mia PaCa2 cells and BxPC-3 cells induced neuritogenesis in the PC-12 cells (Fig 4). Inhibition of secreted NGF in the conditioned media by a neutralizing NGF antibody or inhibition of TRKA activity with the GW441756 inhibitor resulted in a decrease in neuritogenesis from the PC-12 cells (Fig 4). Thus, a reduction in neuritogenesis due to the inhibition of NGF and its receptors points to a reciprocal signaling pathway between Mia PaCa2 and BxPC-3 and the PC-12 cells.

## Conclusions

Several correlative studies have already established a link between increased incidence of PNI and elevated levels of NGF-TRKA signaling pathway proteins [3,4,6,11,12,14,18]. Further, high levels of PNI have also been correlated to pain and dismal prognosis in pancreatic cancer patients [11,13,14,20,36]. Here we have demonstrated that NGF signaling via TRKA between pancreatic cancer cells and surrounding nerves is one of the molecular mechanisms involved in PNI. NGF autocrine signaling via TRKA and perhaps also via p75^NTR^ increases their proliferation and aggressiveness. Paracrine signaling between NGF secreted by the pancreatic cancer cells and TRKA on the surrounding nerves results in increased invasive propensity of the pancreatic cancer cells for these nerves, thereby contributing to PNI. However, more work needs to be done to understand the underlying mechanism of NGF signaling mediated perineural invasion in pancreatic cancer. A deeper understanding of this NGF signaling pathway will aid in designing novel therapeutics that prevent PNI and alleviate pain associated with PNI in patients with pancreatic cancer.

With the availability of TRKA antagonists currently in the clinical trials, a trial specifically designed to use such an antagonist to decrease the onset or worsening of pain for patients with advanced pancreatic cancer is indicated.

## Supporting Information

S1 Fig Inhibition of ERK1/2 phosphorylation by GW441756 in Mia PaCa-2 cells. Cells were treated with GW441756 at the indicated concentrations for 48 hours and cell lysates were analyzed for the indicated proteins using Western blotting. The antibodies for ERK1/2 and pERK1/2 (Thr202/Tyr204) were from Cell Signaling Technology (Danvers, MA) and the antibody for Actin was from Sigma-Aldrich (St. Louis, MO).
(TIF)
