# Supplementary figures and images for: Blocking Nerve Growth Factor Signaling Reduces the Neural Invasion Potential of Pancreatic Cancer Cells
Source: PLoS One. 2016 Oct 28;11(10):e0165586. doi: 10.1371/journal.pone.0165586 (PMC5085053; doi:10.1371/journal.pone.0165586)

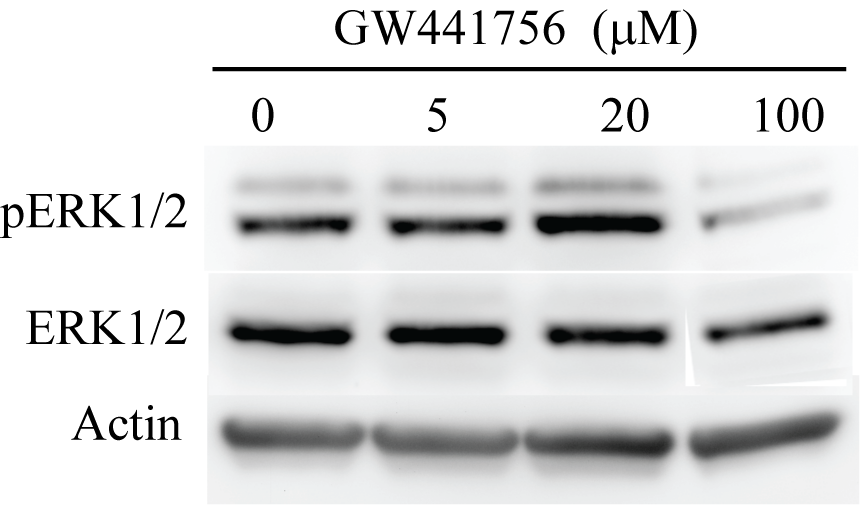

Supplement: S1 Fig — Cells were treated with GW441756 at the indicated concentrations for 48 hours and cell lysates were analyzed for the indicated proteins using Western blotting. The antibodies for ERK1/2 and pERK1/2 (Thr202/Tyr204) were from Cell Signaling Technology (Danvers, MA) and the antibody for Actin was from Sigma-Aldrich (St. Louis, MO). (TIF) [file pone.0165586.s001.tif]
